# Impact of Endemic Besnoitiosis on the Performance of a Dairy Cattle Herd

**DOI:** 10.3390/ani12101291

**Published:** 2022-05-18

**Authors:** Catarina Anastácio, Ricardo Bexiga, Sofia Nolasco, Sara Zúquete, Inês L. S. Delgado, Telmo Nunes, Alexandre Leitão

**Affiliations:** 1 CIISA—Centro de Investigação Interdisciplinar em Sanidade Animal, Faculdade de Medicina Veterinária, Universidade de Lisboa, 1300-477 Lisboa, Portugal; catarina.anastacio@hotmail.com (C.A.); sofianolasco@fmv.ulisboa.pt (S.N.); s.zuquete@edu.ulisboa.pt (S.Z.); inesdelgado@fmv.ulisboa.pt (I.L.S.D.); tnunes@fmv.ulisboa.pt (T.N.); 2 Laboratório Associado para Ciência Animal e Veterinária (AL4AnimalS), 1300-477 Lisboa, Portugal; 3 Escola Superior de Tecnologia da Saúde de Lisboa, Instituto Politécnico de Lisboa, 1990-096 Lisbon, Portugal; 4 Faculdade de Medicina Veterinária, Universidade Lusófona, 1749-024 Lisboa, Portugal

**Keywords:** *Besnoitia besnoiti*, bovine besnoitiosis, dairy cows, serology, clinical signs, dairy production, productive and reproductive parameters

## Abstract

**Simple Summary:**

Bovine besnoitiosis, caused by the Apicomplexa parasite *Besnoitia besnoiti*, has been emerging in Europe as a disease of economic concern to the cattle industry. It is a chronic and debilitating disease mostly reported in beef cattle. However, in Europe, bovine besnoitiosis is increasingly common in dairy cattle; therefore, there is a need to assess the impact of this disease on milk production. To study the effect of *B. besnoiti* infection on dairy production and reproduction, a serological screening was performed on a dairy herd in an endemic area. The results showed that the herd was endemically infected, with high seroprevalence and low clinical prevalence, and the time on herd represented a risk factor to acquire the infection. Seropositive animals and cows with chronic skin lesions revealed higher milk somatic cell counts, and no negative impact on reproductive performance was found.

**Abstract:**

This study aimed to assess the effect of *Besnoitia besnoiti* infection on the reproductive and productive performance of a dairy cattle herd. A serological screening was performed by indirect fluorescent antibody test (IFAT) on every animal aged over one year (*n* = 262). Subsequently, 211 animals were clinically examined, with 96 of those being screened for detection of sclerocysts. The overall seroprevalence was 62.9% (CI95%: 56.1–69.5%). On clinical examination, 7.6% (16/211) of the animals presented chronic skin lesions, and 47.9% (46/96) had sclerocysts. Multivariate logistic regression showed that the time on herd represented a risk factor, and the odds of acquiring the infection increased 1.683× per additional year on herd, ranging from less than a year to 8 years. Seropositivity and the presence of sclerocysts revealed an association with a higher milk somatic cell count, which may have a considerable economic impact on dairy production. Regarding reproductive indicators, no negative impact could be associated with clinical besnoitiosis or positive serological results. In conclusion, our study highlights the need to thoroughly evaluate the economic impact of this emerging disease in dairy herd production to help with decision making at both herd and regional levels, particularly in endemic areas.

## 1. Introduction

Bovine besnoitiosis is a chronic debilitating disease caused by the cyst-forming apicomplexan parasite *Besnoitia besnoiti*, which is closely related to *Toxoplasma gondii* and *Neospora caninum* [1]. The definitive host remains unknown, but cats and other carnivores are suspected [2,3,4,5]. Ruminants, especially cattle, represent the most relevant intermediate hosts, harbouring two different asexual parasitic stages: tachyzoites and bradyzoites, which are responsible for the acute and chronic stages of the disease, respectively [6]. In the acute stage, hyperthermia, oedemas, orchitis, and non-specific clinical signs such as depression, tachypnoea, tachycardia, congestive mucosae, nasal discharge, anorexia, and weight loss are present. The chronic stage is characterized by the presence of tissue cysts and skin lesions such as skin thickening, hyperkeratosis, and alopecia [7]. In bulls, testis atrophy is also common in this stage [7]. However, only a small part of the infected animals manifest clinical signs, with the majority evolving to subclinical infections [8]. Frequently, the occurrence of bovine besnoitiosis coincides with the introduction of newly acquired animals to farms, with seroprevalence rates rapidly increasing in recently infected herds, after the detection of the first clinical case [9,10]. Mechanical transmission either through hematophagous insects, such as *Stomoxys cacitrans* and *Tabanidae*, or iatrogenically by contaminated needles has been experimentally demonstrated [7,11] and is deemed to play an important role at farm level [12].

Bovine besnoitiosis is endemic in tropical and subtropical areas of Africa and in Mediterranean countries such as Portugal, France, and Spain. However, a notable increase of cases and a rising number of affected countries led to its classification in 2010 as an emerging disease in Europe [13]. Since then, several countries of central and western Europe including Italy, Germany, Switzerland, Croatia, Hungary and Belgium were affected [14,15,16], but so were northern regions such as Ireland [17]. The actual impact of the disease remains unknown due to a lack of economic studies, but the consequences are severe in endemic regions [13]. Although considered a neglected parasitic disease with low mortality rates (<10%), the economic impact of bovine besnoitiosis is considered medium to high, with consequences for animal welfare, by veterinary practitioners in endemic areas, according to a study on the perception of bovine besnoitiosis by veterinary field practitioners [18]. In these areas, where morbidity can reach high values (>80%), the economic losses are related to weight loss, poor body condition, decreased milk production that negatively affect calves’ growth, and abortion due to high fever in the acute stages of the disease [12]. Losses are also caused by sterility in bulls due to acute or chronic besnoitiosis [19,20]. The presence of cysts in muscle, connective tissue, fascia, and some organs can lead to partial or even total rejection of carcasses, and hides are of reduced value for tanning [1]. Most of the cases across Europe have been recorded in beef cattle; however, this disease is also present in dairy cattle, mainly in enzootic areas where the majority of animals remain sub-clinically infected [21]. Although decreased productivity in animals with chronic besnoitiosis has been recorded [22], the impact of besnoitiosis on the dairy sector remains largely unknown. The present study aimed to investigate the impact of bovine besnoitiosis on the productive and reproductive performances of a dairy cattle herd. The serological and clinical patterns of the herd and the risk factors associated with *B. besnoiti* infection were also evaluated.

## 2. Materials and Methods

### 2.1. Herd Description and Study Area

The herd in this study was selected because several clinical cases of besnoitiosis had been recorded by veterinary practitioners in the preceding year. This herd had approximately 400 Holstein Frisian cattle, with around 230 lactating cows milked twice a day. The animals were housed but had access to a resting area outside. Heifers and dry cows were also housed but with access to the pasture. Male calves were sold at 2 weeks of age, while female calves were kept on the farm as replacement stock. Additional animals were also bought from surrounding farms with no history of besnoitiosis: three cows in April 2018, six cows in March 2018, and 47 cows in July 2020. The sanitary status of bought-in animals regarding *B. besnoiti* was not known at the time of purchase. A sweeper bull was present in the herd for heifers not pregnant after artificial insemination, however the examination of this animal was not possible during the study. The herd was regularly vaccinated against clostridiosis, infectious bovine rhinotracheitis, bovine viral diarrhea, bovine parainfluenza 3, and bovine respiratory syncytial virus. Deltamethrin was applied to the animals as a pour-on, monthly, during the fly season (March to October). There are three other relevant aspects regarding this particular farm: a history of sharing pastures by accident with beef cattle from neighboring farms, 3 years before; wildlife, particularly foxes and wild boars, are frequently spotted at the farm; the presence of anti-*Neospora caninum* antibodies was detected on the analysis of the bulk tank milk. 

The farm was located in the south of Portugal, near the border with Spain, where bovine besnoitiosis is endemic [23]. This area has a high density of beef cattle under extensive management conditions. The climate is Mediterranean, with warm to hot dry summers and mild to cool wet winters. The average minimum and maximum temperatures are 17 °C and 33 °C, respectively. 

### 2.2. Sampling and Data Collection

All animals included in the study were Holstein Frisian females, aged between 1 and 8 years old, with 79% being born on the study farm and 21% having been bought-in from 3 other farms. The stage of lactation was divided into postpartum (0–35 days), peak lactation (36–90 days), mid lactation (91–305 days), late lactation (>305 days), and dry period, among all animals that had already calved once regardless of age (*n* = 239). The heifer category refers to nulliparous animals included in the sample, aged between 12 and 24 months (*n* = 23). 

Blood samples were collected from the coccygeal vein to dry tubes on 4 separate occasions: October (*n* = 54), November (*n* = 144), December 2020 (*n* = 12), and February 2021 (*n* = 52). Samples were centrifuged (1000× *g* for 20 min), and sera were stored at −18 °C until serological testing. 

A total of 262 animals were sampled and individual data were recorded, namely cattle’s age, origin, sex, and stage of lactation at the respective month of sample collection. Individual data and information about milk production and reproductive indicators were recorded for each sampled animal from farm software (GEA DairyPlan C21 Herd Management Software, Düsseldorf, Germany). 

Reproductive data regarding calving interval, number of inseminations to conception and history of abortion and reproductive disease (metritis and retained fetal membranes) were recorded between January 2020 and February 2021.

Regarding milk production indicators, the data of the sampling month were considered for lactating cows (*n* = 229), whereas, for dry cows (*n* = 10), data from 1 or 2 months prior to the drying-off date were used in the analysis. The indicators analyzed were somatic cell count, daily milk production, 305-mature equivalent milk yield, protein content, and fat content. 

### 2.3. Indirect Fluorescent Antibody Test

Specific antibodies against *B. besnoiti* infection were detected by IFAT. *B. besnoiti* parasites (isolate Bb1Evora03) used in the preparation of IFAT slides were obtained from a naturally infected bovine [8] and were maintained by continuous passages in Vero cell cultures (African Green Monkey kidney epithelial cells), as previously described [24]. To obtain antigen, supernatants from lysed infected cultures were centrifuged to separate tachyzoites from host cell debris at 30× *g* for 5 min, and tachyzoites were pelleted at 800× *g* for 10 min and fixed in phosphate buffered saline containing 1% formalin. IFAT slides were prepared according to Shkap et al. (2002) [25], with slight modifications. Each serum sample was tested in three dilutions: 1:125, 1:250, and 1:500. A positive control from a bovine presenting dermal cysts tested previously by IFAT and a negative control from a non-endemic area were included in each assay. Fluorescein isothiocyanate-labeled, affinity purified sheep anti-bovine immunoglobulines (Serotec AA123F) were used as secondary antibodies, and the screening for results was performed under a fluorescence microscope by two independent observers. The presence of bright fluorescence around the tachyzoites at 1:250 dilution was considered the cut-off for positivity [8].

### 2.4. Clinical Examination

A clinical examination to evaluate the presence of clinical signs ascribable to bovine besnoitiosis was performed in February 2021 on 221 animals.

**Figure 1:**
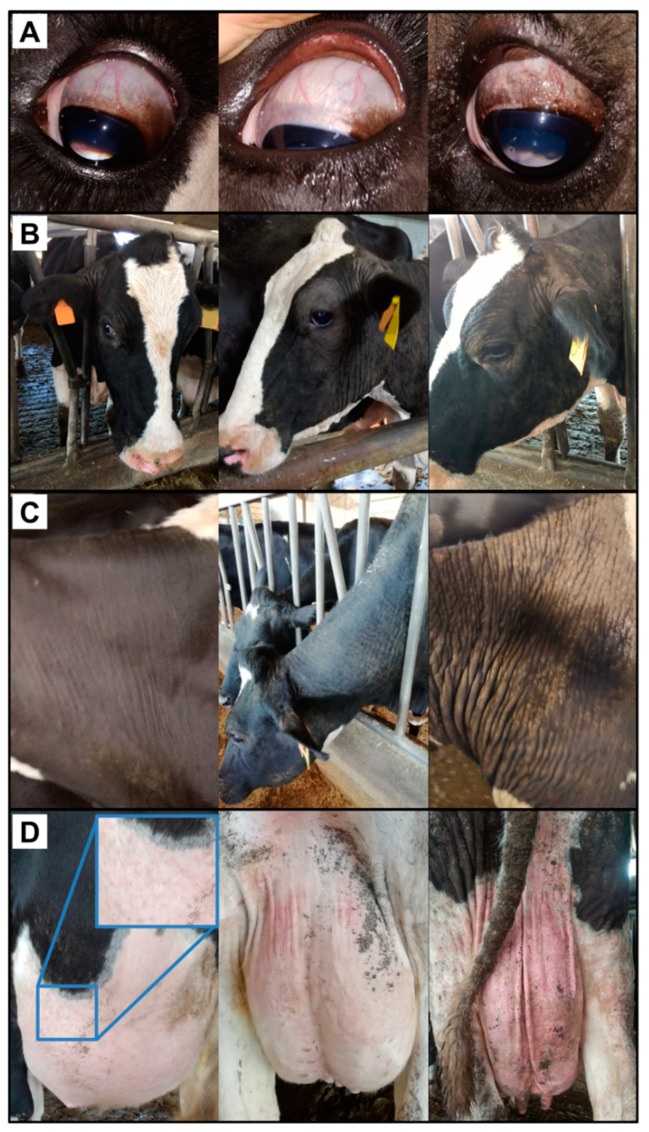
Clinical signs ascribable to bovine besnoitiosis, namely (**A**) presence of cysts in the scleral-conjunctivae and skin appearance alteration in the (**B**) face, (**C**) neck, and (**D**) udder. The clinical findings are ranked by severity from left (less severe) to right (more severe) panels.

In the first approach, animals were systematically examined while housed to check for the presence of chronic skin lesions such as thickening and folding of the skin, hyperkeratosis, alopecia, and presence of cysts in different anatomic areas (face, neck, shoulders, chest, dorsal area, rump, fore and hind limbs, hooves, and udder). Scores 0 (without lesions), 1 (mild), 2 (moderate), and 3 (severe) were assigned to classify the degree of observable lesions for each anatomic area (see Figure 1). For statistical purposes, animals with 5 or more areas with a score ≥1 were considered as clinically affected. Body condition, lameness, ocular or nasal discharge, and other clinical findings were also registered.

In the second approach, during milking, the presence of sclerocysts (Figure 1) were carefully checked. Cows exhibiting cysts on one or both eyes were considered as sclerocyst-positive. Due to the logistic and practical conditions, the ocular area was examined in only 96 cows.

### 2.5. Statistical Analysis

The apparent and real prevalence of tested animals were calculated by EpiTools software Epidemiological Calculators, Ausvet, considering all animals in the study (over 12 months). The Wilson method was used for calculating confidence intervals (CI). Both prevalences were also calculated considering only animals that had already calved once. The productive and reproductive parameters, individual data, clinical observations, and serology results of each animal were entered into Microsoft Excel (Microsoft Excel for Microsoft 365 MSO, Redmond, WA, USA) and analyzed by R^®^ 4.1.1 Software. Qualitative variable analysis was carried out using a Chi-square test. Odds ratios (ORs) were used to evaluate risk factors associated with positivity by IFAT. A logistic regression was performed to evaluate the influence of individual factors (independent variables) on serological results or clinical signs (dependent variables). The quantitative variable analysis was carried out also by a multivariate logistic regression model to ascertain the influence of individual factors with serological results or clinical signs (independent variables) on each reproductive and productive parameter (dependent variables). In both models, among individual factors, only origin, age, and stage of lactation were considered, since sex and breed were the same for all sampled animals.

## 3. Results

The 262 serum samples tested by IFAT for anti-*B. besnoiti* specific antibodies showed that 148 (56.5%) were seropositive. If considering only the animals that had already calved once, the seroprevalence increases to 59.8% (143/239). When adjusted to IFAT sensitivity (89.6%) and specificity (99.7%) [23], the seroprevalence for total sampled animals and for animals that had already calved once were, respectively, 62.9% (95% CI 56.1–69.5%) and 66.7% (95% CI 59.6–73.4%).

Nulliparous and primiparous cows under 2 years old had a lower percentage of positive serological results (20.6%). Animals born on the study farm had a higher percentage of seropositive results (62.0%) compared to animals that had been bought-in from outside farms (42.9%). Multivariate logistic regression, considering serological result as a dependent variable, did not find any significant association between seropositivity and age or origin of animals. However, the time on herd represented a risk factor, and the odds of acquiring the infection increased 1.683× per additional year on herd (Table 1). In agreement, heifers had a lower chance (Odds Ratio = 0.232) of being positive compared to cows in the mid lactation phase (Table 1).

No clinical sign of acute disease was observed; however, 16 cows (7.6%) showed characteristic chronic skin lesions. The body regions where the alterations were more evident to a macroscopic inspection were the face, the neck, and the udder. Upon eye examination, 47.9% (46/96) of the cows presented pathognomonic sclerocysts, of which 89.1% (41/46) were seropositive. Of the five seronegative animals that presented sclerocysts, three also presented skin lesions.

Considering dairy production indicators, seropositive cows had significantly higher milk somatic cell counts (mean value 300,000 cells/mL) than seronegative animals (mean value 189,000 cells/mL) (Table 2). This difference was also observed for animals with sclerocysts, using, as reference, the group of animals not showing cysts (mean values 370,000 cells/mL and 141,000 cells/mL, respectively) (Table 3). In the group of 16 cows presenting skin lesions, this indicator showed the same tendency (mean value 319,000 cells/mL) in comparison with the 195 animals without lesions (mean value 222,000 cells/mL), although not reaching statistical significance (Table 4). The average daily milk production and 305-mature equivalent milk yield were, on average, slightly higher for seropositive cows or those with clinical signs (sclerocysts or skin lesions); however, there was no statistically significant correlation (Table 2, Table 3 and Table 4).

Finally, concerning reproductive performance data, it was observed that animals with chronic skin lesions had a shorter calving interval (Table 4). In the data analyzed, it was not possible to find a statistically significant association between seropositivity or the presence of clinical signs and records of animals with history of abortion (4/5), metritis (26/38), and retained fetal membranes (10/14).

## 4. Discussion

This study reported *B. besnoiti* infection in dairy cattle on a farm located in the south of Portugal. The presence of several chronic clinical cases and a high seroprevalence of 62.9% (95% CI 56.1–69.5%) among tested animals suggest that the herd was endemically infected. Under endemic conditions, the occurrence of acute clinical besnoitiosis tends to be low. However, we admit that the period of the year (October 2020–February 2021) may have contributed to the absence of acute forms in our observations. The prevalence in this farm was in the upper range of the within-herd seroprevalence reported by Waap et al. (2014) [23], who observed a mean within-herd seroprevalence of 33% (95% CI 20.3–46.0%) among positive cattle herds in Portugal. However, most farms in that study were under extensive management conditions, and no dairy cattle herds were found to be positive. Seroprevalence in this herd may have been higher due to the intensive management conditions, with higher animal density and closer cohabitation with infected cattle, which may favor a more efficient transmission promoted by direct contact between animals [9,17]. Although the status of the sweeper bull in relation to the *B. besnoiti* infection is unknown, in previous studies, the relevance of this transmission route revealed to be limited [26]. Biting flies may have also played a role through mechanical transmission of *B. besnoiti* to and within this herd [9,26]. However, there is a lack of epidemiological studies in dairy cattle to further substantiate this possibility. Despite being described in a high variety of breeds of both dairy and beef cattle [13], bovine besnoitiosis occurrence seems to be less frequent in dairy cattle, as shown by cross sectional studies [23,27,28]. According to cattle individual data, older animals or cattle that were born on the study farm had a higher percentage of seropositive results compared to bought-in animals, which might be related to a longer cohabitation period with infected animals, since vertical transmission of *B. besnoiti* was never reported and there are claims that it does not occur [26]. The time on herd represented a risk factor for *B. besnoiti* seropositivity, since animals that were the longest in the herd had a higher chance of being infected. This seems to support that *B*. *besnoiti* transmission occurs horizontally, in agreement with previous studies on beef cattle reared under extensive conditions [29]. The risk of exposure to *B. besnoiti* may increase when susceptible cattle remain housed together with infected animals.

Considering animals with chronic skin lesions, the clinical prevalence was 7.6%, corresponding to values reported in endemically infected areas (1–10%), where most of infections are subclinical [1]. The distribution of skin lesions in those dairy cows was similar to what has been reported in infected beef cattle. Body regions with higher parasitic loads, as evaluated by PCR, include limbs (especially the distal parts of the hindlegs), rump, face, eyes, and muzzle [30], and all commonly present skin lesions. However, despite not being reported as so densely parasitized, we observed in this study that the face, the neck, and the udder were the areas presenting more pronounced macroscopic changes, and, therefore, these areas should be privileged at clinical examination.

Regarding the impact of a *B. besnoiti* infection on milk production and quality, statistical analysis revealed that seropositivity and the presence of sclerocysts were associated with increased somatic cell count. Parasite excretion in milk could be considered; however, this hypothesis has been previously discarded by the absence of *B. besnoiti* DNA in the colostrum from infected cows [26]. It was not possible to assess the possibility of a correlation between somatic cell count and a clinical mastitis in the scope of this study.

As reviewed by others [1,12], *B. besnoiti* infection may be responsible for a decrease in milk production. However, a recent study evaluating milk production in an infected dairy herd found no statistical evidence of an effect of seropositivity or of the presence of clinical signs on milk production, despite some cows with skin cysts presenting a decrease in certain productive parameters such as daily milk production, fat and protein content, and mature equivalent milk yield [22]. Ryan et al. (2016) [17] report that, in the acute phase, an initial milk drop syndrome in animals with pyrexia and limb/joint swelling can occur and that some of the chronically infected animals can recover, at least partially, with milk production returning to optimal levels. In the present study, seropositive animals (including asymptomatic ones) presented, on average, higher daily productions and 305-mature equivalent milk yields. The difference was not statistically significant, but the observation does not give support to a reduction of milk production caused by *B. besnoiti* infection, highlighting the necessity of a better characterization of the impact of besnoitiosis in dairy cattle herds. 

In terms of reproductive indicators, the calving interval was significantly shorter in animals with chronic besnoitiosis skin lesions. This observation was not expected and may suggest that animals with higher performances are more susceptible to *B. besnoiti* infection. Seropositivity or the presence of clinical signs did not show any association with history of abortion and reproductive disease. Although the presence of the parasite in the reproductive tract of cows has already been reported [31], the impact of bovine besnoitiosis on cow fertility remains unknown. Still, hyperthermia during the acute phase of the disease may act as a potential cause of pregnancy loss.

The source of infection in this endemically infected herd was unclear and should be investigated to base effective control measures. It is of note that the seroprevalence among animals originating from outside farms was lower, and the time on herd represented a risk factor, suggesting that the origin of the focus is related to the studied farm. Bovine besnoitiosis is endemic in the south of Portugal [23], and such an epidemiological scenario is compatible with the presence of a definitive host, abundance of reservoirs, or activity of biting insects [1]. 

There is no restriction to animal trade associated with bovine besnoitiosis in Europe, except in Switzerland, where it is a notifiable disease (Ordonnance sur les épizooties 916.401 du 27 juin 1995, Etat le 1er janvier 2022, Section 10). However, transmission of the parasite between herds should be avoided by biosecurity and biocontainment measures, given the economic impact of the disease. Serological screening is recommended for any new entrances in the herd. It is of note, however, that we observed scleroderma signs in five seronegative cows. For four of these animals, sampling took place 3 to 4 months before the clinical examination, but one of these seronegative cows was sampled on the same day as clinical examination. So, as reported by others [6,32], a negative serological result does not guarantee the absence of *B. besnoiti* infection and a protocol combining laboratorial and clinical methods should be considered.

The control of bovine besnoitiosis is difficult, and measures such as the culling of seropositive animals, animals with severe clinical signs, or even limited to animals considered super-spreaders [33] have substantial impact on a farm. Our results highlight the need to evaluate the economic impact of the disease on dairy herd production systems to assist in the adoption of more adequate measures at herd and regional levels.

## 5. Conclusions

In conclusion, this herd was endemically infected with high seroprevalence, but only a few animals were clinically affected. Our results demonstrate that seroprevalence may be high in endemic areas, even in dairy cattle, and high somatic cell counts could be associated with infection. However, neither reduction in milk production nor increased calving intervals could be associated with *B. besnoiti* infection. This study was based on a single herd, and more studies of reproductive and dairy production parameters are required to determine the real impact of bovine besnoitiosis and, therefore, to assist taking decisions for control of the disease. A control program should be adapted to each herd or region according to its particular epidemiological scenario and should be based on the early detection of infection by a standardized combination of clinical and laboratory tests, reinforcement of biosecurity management measures, and on information sharing among farmers and veterinarians on the biological aspects of besnoitiosis, on the consequences to the animal health and welfare, and on its economic impact, especially in re-emergency areas.

## Figures and Tables

**Table 1:** Seroprevalence of *Besnoitia besnoiti* antibodies by individual data (age, origin, and stage of lactation) and clinical findings (skin lesions and sclerocysts) using multivariate logistic regression.

Variable	Category	Number	IFAT+	(%)	OR	*p*
Age (years)	1	34	7	20.6	Continuous 0.837	0.394
	2	72	40	55.6		
	3	49	33	67.3		
	4	47	28	59.6		
	5	39	19	65.5		
	6	13	9	69.2		
	7	14	9	64.3		
	8	4	3	75.0		
	Total	262	148	56.5		
Time on herd (years)	<1	47	18	38.3	Continuous 1.683	<0.05
	1	34	7	20.6		
	2	75	43	57.3		
	3	43	29	67.4		
	4	21	15	71.4		
	5	20	18	90.0		
	6	12	9	75.0		
	7	7	6	85.7		
	8	3	3	100.0		
	Total	262	148	56.5		
Animal’s origin	Study farm	206	124	60.2	reference 1.860	0.494
	Outside farms	56	24	42.9		
	Total	262	148	56.5		
Stage of lactation	Mid lactation	112	67	59.8	reference 1.426 1.015 0.505 0.994 0.232	0.373 0.984 0.111 0.988 <0.05
	Late lactation	46	32	69.6		
	Dry period	10	6	60.0		
	Postpartum	33	15	45.5		
	Peak lactation	38	23	60.5		
	Heifer	23	5	21.7		
	Total	262	148	56.5		

**Table 2:** Descriptive statistics (mean, standard deviation, minimum, and maximum) of productive and reproductive parameters by serological results with simple linear regression.

Variable	Group	Number	Mean (σ)		Min.	Max.	Estimated	*p*
Milk somatic cell count (×1000 cells/mL)	Seronegative	96	189	(266)	18	1889	reference 139.63	<0.05
	Seropositive	143	300	(611)	10	5038		
	Total	239	255	(505)	10	5038		
Daily milk production (kg)	Seronegative	96	34.0	(8.7)	10.3	56.8	reference 1.08	0.280
	Seropositive	143	34.9	(8.2)	15.2	56.6		
	Total	239	34.6	(8.4)	10.3	56.8		
305-mature equivalent milk yield (kg)	Seronegative	96	10,403	(1635)	5866	14,802	reference −62.6	0.767
	Seropositive	143	10,710	(1674)	7487	15,613		
	Total	239	10,587	(1665)	5866	15,613		
Milk protein content (%)	Seronegative	96	3.37	(0.29)	2.69	4.02	reference −0.01	0.910
	Seropositive	143	3.38	(0.34)	2.62	4.56		
	Total	239	3.38	(0.32)	2.62	4.56		
Milk fat content (%)	Seronegative	96	4.38	(0.86)	1.97	7.62	reference 0.17	0.126
	Seropositive	143	4.48	(0.86)	2.28	6.72		
	Total	239	4.44	(0.86)	1.97	7.62		
Calving interval	Seronegative	59	434	(98)	324	885	reference 1.86	0.916
	Seropositive	103	438	(106)	323	912		
	Total	162	436	(103)	323	912		
Number of inseminations	Seronegative	61	2.8	(1.8)	1	8	reference 0.14	0.655
	Seropositive	110	2.9	(2.0)	1	10		
	Total	171	2.8	(1.9)	1	10		

**Table 3:** Descriptive statistics (mean, standard deviation, minimum, and maximum) of productive and reproductive parameters by absence or presence of sclerocysts with simple linear regression.

Variable	Group	Number	Mean (σ)		Min.	Max.	Estimated	*p*
Milk somatic cell count (×1000 cells/mL)	Without cysts	50	141	(141)	20.3	712	reference 304.27	<0.05
	Sclerocysts	46	370	(820)	14	5038		
	Total	90	252	(590)	14	5038		
Daily milk production (kg)	Without cysts	50	35.4	(6.4)	20.8	50.4	reference 1.65	0.261
	Sclerocysts	46	37.4	(8.7)	15.2	56.8		
	Total	90	36.4	(7.6)	15.2	56.8		
305-mature equivalent milk yield (kg)	Without cysts	50	10,306	(1244)	7949	13,604	reference 316.8	0.296
	Sclerocysts	46	10,982	(7815)	7815	14,433		
	Total	90	10,633	(1516)	7815	14,433		
Milk protein content (%)	Without cysts	50	3.33	(0.27)	2.62	4.02	reference 0.04	0.487
	Sclerocysts	46	3.35	(2.72)	2.72	4.16		
	Total	90	3.34	(0.29)	2.62	4.16		
Milk fat content (%)	Without cysts	50	4.59	(0.81)	2.46	6.65	reference 0.11	0.480
	Sclerocysts	46	4.64	(3.10)	3.11	6.72		
	Total	90	4.61	(0.82)	2.46	6.72		
Calving interval	Without cysts	50	403	(70)	326	584	reference 17.77	0.350
	Sclerocysts	46	423	(79)	328	607		
	Total	90	413	(75)	326	607		
Number of inseminations	Without cysts	50	2.3	(1.8)	1	8	reference 0.15	0.700
	Sclerocysts	46	2.6	(1.6)	1	7		
	Total	90	2.5	(1.7)	1	8		

**Table 4:** Descriptive statistics (mean, standard deviation, minimum, and maximum) of productive and reproductive parameters by absence or presence of skin lesions with simple linear regression.

Variable	Group	Number	Mean (σ)		Min.	Max.	Estimated	*p*
Milk somatic cell count (×1000 cells/mL)	Without lesions	195	222	(483)	11	5038	reference 113.16	0.395
	Skin lesions	16	319	(517)	14	2223		
	Total	211	230	(488)	11	5038		
Daily milk production (kg)	Without lesions	195	35.0	(8.0)	10.3	56.6	reference 0.01	0.997
	Skin lesions	16	36.9	(9.4)	23.4	56.8		
	Total	211	35.2	(8.2)	10.3	56.8		
305-mature equivalent milk yield (kg)	Without lesions	195	10,629	(1598)	6267	15,613	reference −605.0	0.134
	Skin lesions	16	10,555	(1834)	7815	14,433		
	Total	211	10,622	(1619)	6267	15,613		
Milk protein content (%)	Without lesions	195	3.37	(0.30)	2.62	4.43	reference 0.01	0.896
	Skin lesions	16	3.30	(026)	2.77	3.86		
	Total	211	3.36	(0.30)	2.62	4.43		
Milk fat content (%)	Without lesions	195	4.44	(0.87)	1.97	7.62	reference 0.13	0.550
	Skin lesions	16	4.62	(0.83)	0.83	6.16		
	Total	211	4.46	(0.86)	1.97	7.62		
Calving interval	Without lesions	195	431	(96)	324	912	reference −69.35	<0.05
	Skin lesions	16	365	(31)	332	424		
	Total	211	425	(94)	324	912		
Number of inseminations	Without lesions	195	2.7	(1.8)	1	10	reference −1.08	0.061
	Skin lesions	16	1.6	(0.9)	1	3		
	Total	211	2.6	(1.8)	1	10		

## Data Availability

The data presented in this study are available upon request from the corresponding authors.
